# Investigating purchasing preferences of cannabis products on social media: conjoint analysis and online survey

**DOI:** 10.1186/s42238-026-00478-w

**Published:** 2026-07-29

**Authors:** Michael Robert Haupt, Joshua S. Yang, Matthew Nali, Tim Ken Mackey

**Affiliations:** 1 Global Health Program, Department of Anthropology, University of California - San Diego, 9500 Gilman Drive, MC:0505, La Jolla, San Diego, CA 92093 USA; 2 Global Health Policy & Data Institute, San Diego, CA USA; 3 Department of Public Health, California State University, Fullerton, CA USA; 4 S-3 Research LLC, San Diego, CA USA

**Keywords:** Cannabis purchasing, Cannabis attitudes, Social media, Online advertising, Online drug sourcing, Social networking sites (SNS), Conjoint analysis, Demographics, Branding preferences

## Abstract

**Background:**

The proliferation of cannabis advertising on social media platforms indicates a need to understand the influence of online marketing strategies on purchasing and use patterns. This study uses conjoint analysis and survey measures to quantify and rank influential features of social media ads on intention to purchase cannabis products. We also conduct exploratory analysis to identify demographics and attitudes associated with cannabis branding preference and likelihood of purchasing product from a high-risk channel.

**Methods:**

Participants were recruited from the Prolific panel in December 2024. Inclusion criteria were self-report requirements for: (1) having previously shopped online; and (2) having used cannabis either medicinally or recreationally. Mock social media posts that advertised cannabis products were tested using a choice-based conjoint (CBC) exercise and hierarchical regression modeling. Follow-up survey questions were used to measure purchasing habits and influences on cannabis perceptions, and regression analysis was conducted to examine predictors of branding preferences and high-risk cannabis purchasing.

**Results:**

Two hundred fifty-three participants were recruited. In the conjoint analysis, *Brand Display* was the highest ranked attribute on purchasing preference (56.73, SD = 15.42) while *Platform* ranked lowest (6.91, SD = 5.30). Within the attribute *Brand Display*, non-celebrity branded was the highest ranked level (91.24, SD = 95.36) followed by unbranded product displayed on a table (51.55, SD = 67.37). Other high ranking post features include: having a store as the author (compared to individuals) (16.58, SD = 42.56), mentioning Indica effects in caption (9.43, SD = 29.91), being on X (formerly Twitter) compared to Instagram, (3.14, SD = 21.57) and showing high engagement (i.e., likes and comments) (9.89, SD = 23.59). Regression analysis showed that educational level (*p*<.001), age (*p*<.001), and race (*p*<.05) had significant associations with cannabis branding preferences. Education was also negatively associated with ever making a high-risk purchase (*p*<.001) while age showed a positive association (*p*<.05).

**Conclusions:**

Branding was the most important factor in purchasing intention among cannabis online consumers and results from both the conjoint analysis and survey show that cannabis users are most interested in relaxation effects and do not value celebrity endorsements. These findings can inform future studies in identifying and characterizing online marketing promotion that is influential on consumer cannabis use and sourcing behavior.

**Supplementary Information:**

The online version contains supplementary material available at https://doi.org/10.1186/s42238-026-00478-w.

## Introduction

Greater public acceptance of cannabis within the United States that began in the 1990s has been followed by increasing legalization of both medicinal and recreational use (Chiu et al. 2022). With the easing of federal cannabis law enforcement since 2009 (Caulkins 2024), thirty-nine states, three territories and the District of Columbia allow the medical use of cannabis products as of February 2025; twenty-four states, three territories and the District of Columbia allow or regulate cannabis for adult recreational use (National Conference of State Legislatures 2025). The liberalization of attitudes and state policies toward cannabis have also been paralleled by increasing in use – including daily and near daily use, days of use per capita, past month prevalence, and past year prevalence (Caulkins 2024)– as well as growth in the cannabis market and spending on cannabis, most notably for adult recreational use (Hutchison et al. 2019).

Increasing diversity in the number and types of products have accompanied market growth (Spindle et al. 2019), including variation in chemotypes, formulations, and methods of administration. One typology of cannabis products sold online, for example, categorized over 500,000 products into 27 product categories across five routes of administration (Nali et al. 2024). Retailers utilize a variety of themes in their marketing to enhance the appeal of cannabis products (e.g., product design, images of users exploring nature, messaging that emphasizes convenience)(Chen-Sankey et al. 2025, Rhee and Jung 2023) as well as marketing strategies, such as lack of age verification, use of social media, health benefit claims, and offering price promotions (Cui et al. 2024, Nali et al. 2025). The proliferation of product types and retailer marketing activities has created a gap between the products people use and what is known about their health impacts (Hutchison et al. 2019). This knowledge gap is attributed to policy and institutional barriers, where adult use (recreational) cannabis’ schedule I status limits available legal supply used for research, restricts federally funded institutions from conducting research, and makes it difficult to receive an exemption from the US Food and Drug Administration (FDA) to study cannabis products now widely used in state-legalized markets (Hutchison et al. 2019).

In the absence of a coherent regulatory infrastructure for cannabis products, it is critical to understand consumer product preference as they navigate the cannabis marketplace. One qualitative study found that factors such as price, quality, packaging and warnings, the source of the cannabis, and social influences may influence product preferences (Donnan et al. 2022), while a best-worse scaling experiment found that quality, strain type, price, THC (tetrahydrocannabinol) level, and whether a product was pesticide-free were among the most important product attributes among a scale of 20 characteristics (Zhu et al. 2021). Discrete choice experiments (DCE) have found that: THC potency is a primary factor in cannabis edible product choices (Donnan et al. 2024), THC potency and price are influential factors for cannabis vape product choices (Donnan et al. 2023), and potency and package information are the most important factors among numerous product attributes for dried flower cannabis choices (Donnan et al. 2023).

Though choice experiments have been used to investigate appealing features of cannabis products, they have not been used to identify influential marketing strategies used on social networking sites (SNS). Further, while previous analysis of cannabis marketing provide a foundation on general thematic areas in social media and other online channels (Chen-Sankey et al. 2025, Rhee and Jung 2023, Cui et al. 2024, Soleymanpour et al. 2021), there is no work examining influential features of cannabis advertisements within the context of social media marketing, such as the image used in posts (e.g., branded product vs. unbranded), seller characteristics as shown in a profile picture (e.g., individuals vs. stores), product description in the post captions (e.g., mentioning effects such as feeling relaxed), and contextual properties of posts (i.e., platform, number of likes/comments received).

While it is possible to find contaminates that pose health-risks in state-regulated cannabis products (Jameson et al. 2022), this risk is exacerbated when purchasing from unregulated channels such as unlicensed dealers or black markets due to lack of transparency and accountability (Lupkin and Mann 2025)(Suraev et al. 2022). The risks of purchasing from unregulated sellers are further amplified for online transactions, where accountability can be almost impossible to obtain due to lack of identity verification when creating profiles. Despite these risks, motivations for purchasing cannabis from marketing and promotional materials that link to illicit and high-risk sources remain understudied. Previous research identifies price as a main factor for illicit cannabis sourcing (Fataar et al. 2021, Robertson and Thyne 2021), however, there is no work examining how personal factors such as demographic characteristics (e.g., age, race, education level) and attitudes towards illicit cannabis influence the likelihood of purchasing product from an unregulated channel. This work intends to fill these gaps in the literature.

Hence, the purpose of this study was to use a discrete choice experiment (Choice Based Conjoint) to quantify and rank influential features of social media ads promoting THC-based cannabis products. We focused on THC-based products because they produce psychoactive effects, generally pose more potential health risks (e.g., Cannabis Use Disorder, Cannabinoid Hyperemesis Syndrome, etc.), and may be subjected to consumer confusion due to changing definitions of hemp (e.g., contains a total THC concentration of no more than 0.3% on a dry-weight basis). Since cannabis marketing for both licit and illicit products is prominent across online marketplaces and social media platforms (Nali et al. 2024, Nali et al. 2025, Soleymanpour et al. 2021, Anderdal Bakken and Kirstine Harder 2023, Asquith 2021, Jenkins et al. 2021, Marinello 2024, Rup et al. 2020), and engagement with content has the potential to influence cannabis use (Trangenstein et al. 2019, Whitehill et al. 2020), this study aims to characterize persuasive elements of cannabis advertising while accounting for factors unique to social networking sites. Our study builds on previous work that uses conjoint analysis to rank features of drug-selling social media posts that are most influential on safety perceptions (Haupt et al. 2024). This study also uses multivariate regression modeling and principle component analysis (PCA) to explore how cannabis attitudes and user demographics influence cannabis branding preferences and the likelihood of purchasing from a high-risk channel (e.g., unauthorized seller on social networking site).

### Overview of conjoint analysis

We used conjoint analysis to assess features of social media posts advertising THC-based cannabis products that are most influential on intent to purchase. Conjoint analysis is an experimental approach developed in the field of mathematical psychology and is often employed by market researchers to quantify preferences for products among consumers (see (Green et al. 2004, Green and Srinivasan 1990) for a more detailed review). A conjoint study mimics complex decision-making processes that require people to “consider jointly” multiple attributes of a product by choosing hypothetical alternatives. When designing a conjoint exercise, a product is thought of as being comprised of attributes (e.g., Color) that has several possible levels (e.g., Blue, Red). Respondent preferences are assessed using part-worth utility scores based on the levels chosen most often in the conjoint exercise.

The use of conjoint analysis for examining preferences of drug products has grown in recent years (Al-Omari et al. 2022). Pharmacology researchers and medical scientists have used conjoint analysis to evaluate patient preferences for products such as HIV prevention medication (Shrestha et al. 2018) and vaccine treatments (Sun et al. 2020). Recent work has extended the use of conjoint to examine illicit drug advertising on social media and tests post features that convey safety such as drug offerings (e.g., Adderall, cocaine, LSD), profiler picture of seller, and use of emojis (Haupt et al. 2024).

An important advantage of conjoint analysis is that it can reduce social desirability bias in survey responses and identify covert attitudes (Caruso et al. 2009, Horiuchi et al. 2022, Korn et al. 2020). Since the present study requires participants to disclose sensitive information related to drug purchasing preferences, conjoint analysis was used to mitigate potential concerns from social desirability biases. While conjoint analysis has previously been used to examine preferences of cannabis products (Donnan et al. 2024, Donnan et al. 2023, Donnan et al. 2023), there is currently no work that uses this approach to assess cannabis purchasing preferences within the context of social media.

### Present study

This study uses conjoint analysis to assess influential features of social media posts on intention to purchase cannabis products. Table 1 shows the tested attributes and levels in the conjoint exercise, which were identified as influential features of social media posts in prior studies (Haupt et al. 2024, Cheung et al. 2022, Rauschnabel et al. 2012): *Brand Display*, *Seller*, *Description*, *Platform*, and *Post Engagement*. See Table 1 for specific hypotheses tested for each attribute and whether they were supported in the present study.

**Table 1:** Hypotheses for tested attributes and levels of social media cannabis ads

Attribute	Levels	Hypothesis
*Brand* *Display*	Celebrity Brand	*H1*: Brand Display would be the highest ranked attribute Fully Supported *H2*: Posts with branded products will rank higher than unbranded Partially Supported: Non-Celebrity brand was ranked highest, however, Unbranded – Plain ranked higher than Celebrity Branded.
	Branded (Non-Celebrity)	
	Unbranded – Consuming	
	Unbranded – Plain	
*Seller*	Individual – White Male	*H3*: Posts with the Store as author will rank higher than individuals Fully Supported
	Individual – Black Female	
	Store	
*Description*	Ad Only	*H4*: Posts mentioning Sativa and Indica effects will rank higher than Ad Only and Ad + Personal Endorsements Partially Supported While level preferences corresponded with the expected rankings, Chi square test did not show a statistically significant differences between levels.
	Ad + Personal Endorsement	
	Ad + Sativa Effect	
	Ad + Indica Effect	
*Platform*	Instagram	*Literature is insufficient to propose a hypothesis*
	Twitter	
*Post Engagement*	Low	*H5*: Posts with high engagement will rank higher than low. Fully Supported
	High	

The attribute *Brand Display* tests the extent that cannabis branding influences intent to purchase. This attribute was tested because brand imagery encourages consumers to draw inferences about a product, as demonstrated in prior work showing that branded packaging can create positive product associations and increase appeal for cannabis products (Leos-Toro et al. 2021, Goodman et al. 2021). We tested two branded levels: *Celebrity Brand* and *Branded (Non-Celebrity)*. Since many celebrities in recent years have started cannabis brands (e.g., U.S. hip hop artist Snoop Dog) (Gibson 2021), the Celebrity brand level assesses the impact of celebrity affiliation on purchase intent compared to non-celebrity brands. Two levels for unbranded cannabis products were also tested: *Unbranded – Consuming*, which displays unbranded cannabis products being consumed by people, and *Unbranded – Plain*, which displays unbranded cannabis flower laying on a table. Since imitation among peers is a consistent influence for smoking behaviors (Harakeh et al. 2007, Hoek et al. 2022), the *Unbranded – Consuming* level tests conformity effects by displaying people smoking cannabis products in social settings. The *Unbranded – Plain* level serves as a control comparison and displays cannabis flower by itself with no packaging or imagery of people using a product. Our unbranded levels differ from previous work that only tested “plain” packaging (Leos-Toro et al. 2021, Goodman et al. 2021) since we display cannabis outside of product packaging, which corresponds to imagery used in social media settings. We proposed two hypotheses for this attribute:

- H1: Brand Display would be the highest ranked attribute
- H2: Posts with branded products will rank higher than unbranded

The *Seller* attribute tests whether cannabis users are more likely to purchase from individual sellers compared to an official store. Prior work indicates that cannabis users tend to prefer purchasing from storefronts over products sold from individual sellers (Xing and Shi 2024, Bradley et al. 2024). In order to account for perceived differences from gender and race of individual sellers, we tested two levels for individuals: *White male* and *Black female*. These groups were tested because previous work shows gender and race are associated with differences in cannabis use patterns (Hasin et al. 2019, Jeffers et al. 2021). In the present study, we examined if these demographic differences in cannabis use extended to consumer purchasing preferences from sellers. Since we were unable to find literature examining effects from demographic characteristics of sellers, we did not propose a hypothesis for preference differences between individual sellers. However, we tested the following hypothesis on preference for store vendors based on the literature:

- H3: Posts with the Store as author will rank higher than individuals

The *Description* attribute tests the impact of the post caption on purchase intent. The *Ad Only* level mentions that cannabis products are available for purchase with no other description while *Ad + Personal Endorsement* tests the effect from including a personal experience of using the product (e.g., these joints are so good! ). The remaining 2 levels test the effects from mentioning effects from Sativa (e.g., energizing) and Indica (e.g., relaxing) strains. These levels were selected because cannabis users tend to show situational preferences when using Sativa (e.g., during the day, at a party) compared to Indica (e.g., before going to bed) (Sholler et al. 2022). Since health benefits are often included in cannabis marketing (Cui et al. 2024), we hypothesize that:

- H4: Posts mentioning Sativa and Indica effects will rank higher than Ad Only and Ad + Personal Endorsements

The *Platform* attribute tests the impact of social media platform on purchase intent, as prior work reveals that advertising tactics for drug sellers can vary due to differences in platform features (e.g., word count limits)(Haupt et al. 2022). Instagram and X (formerly Twitter) were chosen due to predominance of cannabis content and being frequently used for cannabis advertising and selling (Soleymanpour et al. 2021, Anderdal Bakken and Kirstine Harder 2023, Asquith 2021, Jenkins et al. 2021). Since we were unable to find existing work examining platform effects on cannabis purchasing intent, we did not propose a hypothesis for this attribute.

The *Engagement* attribute tests the impact of post engagement on intent to purchase. Since attention on social media resembles a power law distribution, where the vast majority of engagement is concentrated among a small fraction of highly active users (Wihbey 2014), posts in the low engagement level have between 1 and 10 likes and 1–5 comments/retweets to correspond to the average engagement rates received on posts from typical user accounts (Express A., 2025). Posts in the high engagement level have 100–500 likes and 50–250 comments/retweets, which correspond to typical engagement received for posts from larger accounts representing international brands (Vries et al. 2012). Since higher engagement would signal social endorsement from other users, we hypothesize that:

- H5: Posts with high engagement will rank higher than low engagement.

After completing the conjoint exercise, participants were asked additional questions to measure their attitudes on licit and illicit cannabis use and preferences for product features (e.g., offers health benefits, lower price). These questions were adapted from surveys used in previous work on drug purchasing attitudes (Haupt et al. 2024, Moureaud et al. 2021). We also asked questions about demographic characteristics pertaining to age, race, gender, education level, income, and urbanicity, as prior research shows these factors are associated with cannabis use and purchasing preferences (Hasin et al. 2019, Jeffers et al. 2021, Han et al. 2017, Lim et al. 2022, Maillet 2024). Exploratory analysis using multivariate regression modeling was then conducted to examine the influence of cannabis attitudes and demographic factors on cannabis branding preference, as measured from the conjoint exercise, and the likelihood of purchasing product from a high-risk channel (e.g., unlicensed seller on SNS).

## Methods

This study is divided into two parts. In part 1, participants completed the conjoint exercise. In part 2 respondents answered questions about cannabis purchasing preferences, attitudes towards licit and illicit products, and marketing and information sources.

### Data collection

Participants were recruited from Prolific during December 2024. Prolific is a platform designed to improve on existing crowdsourcing sites used for online recruiting and generally outperforms other popularly used sample sources in data quality (Douglas et al. 2023, Peer et al. 2022). Participants were eligible if they reported having previously shopped online, and have used cannabis either medicinally or recreationally in the past year. Since Prolific does not have these criteria as preset screeners, we used custom screening questions to determine eligibility. The program Lighthouse Studios developed by Sawtooth Software was used to confirm that a sample size of *n* = 250 has sufficient statistical power to observe main effects from tested conjoint design. Participants were removed if they failed at least 2 of the following data quality checks: failing attention checks, straight lined survey questions, provided nonsensical responses to open text questions, and completed the survey at an unusually quick time (10th percentile or less). We initially had *n* = 5 participants over our target due to Prolific’s screening procedures. After filtering for data quality, the final sample size was *n* = 253. The conjoint exercise was created and hosted on Lighthouse Studio and the survey was created on Qualtrics. Data analysis was conducted in R and SPSS. All supplementary materials, screener questions, full conjoint analysis results, and a report on the statistical power of the conjoint design for the present study can be found at the following Open Science Framework (OSF) depository: https://osf.io/ur6ma/?view_only=673851dec544422485342b590fd36511.

IRB approval was obtained from University of California, San Diego (#810321) and all methods and protocols were carried out in accordance with IRB guidelines. Prolific participants completed the survey anonymously and were compensated based on standard survey-taking rates on the platform. Informed consent was obtained from all participants.

### Conjoint design

A total of 192 mock posts were created based on every possible combination of tested attributes (*Brand Display* (4 levels) x *Seller* (3 levels) x *Description* (4 levels) x *Platform* (2 levels) x *Post Engagement* (2 levels) = 192). Images for *Brand Display* levels were selected based on similarity to images used in real cannabis-related content on social media. In the Celebrity Brand level, the following product brands were selected based on high popularity and publicity: Seth Rogan, Snoop Dog, and Woody Nelson. Celebrity images were selected from real promotional material where the celebrity is featured with their product, and 5 different versions were used for each celebrity to account for variations in marketing visuals. In the Non-celebrity Branded level, 3 different cannabis brands were selected from posts on their social media accounts that advertise cannabis flower or pre-rolled joints with 5 versions each to account for variation. Images in the *Unbranded – Consuming* and *Unbranded – Plain levels* were selected from online search with 5 versions of each level to account for visual variation.

For the *Seller* attribute, images for individual user levels (White Male and Black Female) were selected based on similarity to real influencer accounts involved in cannabis-related discourse. To account for variations in appearance from individuals, 5 different versions of images were used for each level. Images for the Store level were logos from 5 real cannabis dispensaries.

Caption text used for the *Description* attribute was based on real world cannabis marketing. For the Ad Only level, the caption text mentioned the brand selected for *Brand Display* is available for purchase. For posts where *Brand Display* is unbranded, the caption mentions the featured product (e.g., flower). Text used for levels mentioning Personal Endorsements, Sativa Effects, and Indica Effects were based on marketing language used in real cannabis-related posts. For all *Description* levels, 5 different versions were used to account for variations in wording.

In order to test realistic stimuli, posts were created using an online mock social media post generator (zeoob.com). For the *Platform* attribute, we had the generator create posts using the user interface for Instagram and Twitter. For *Post Engagement*, we were able to input engagement metrics based on low and high ranges for each level.

**Fig. 1:**
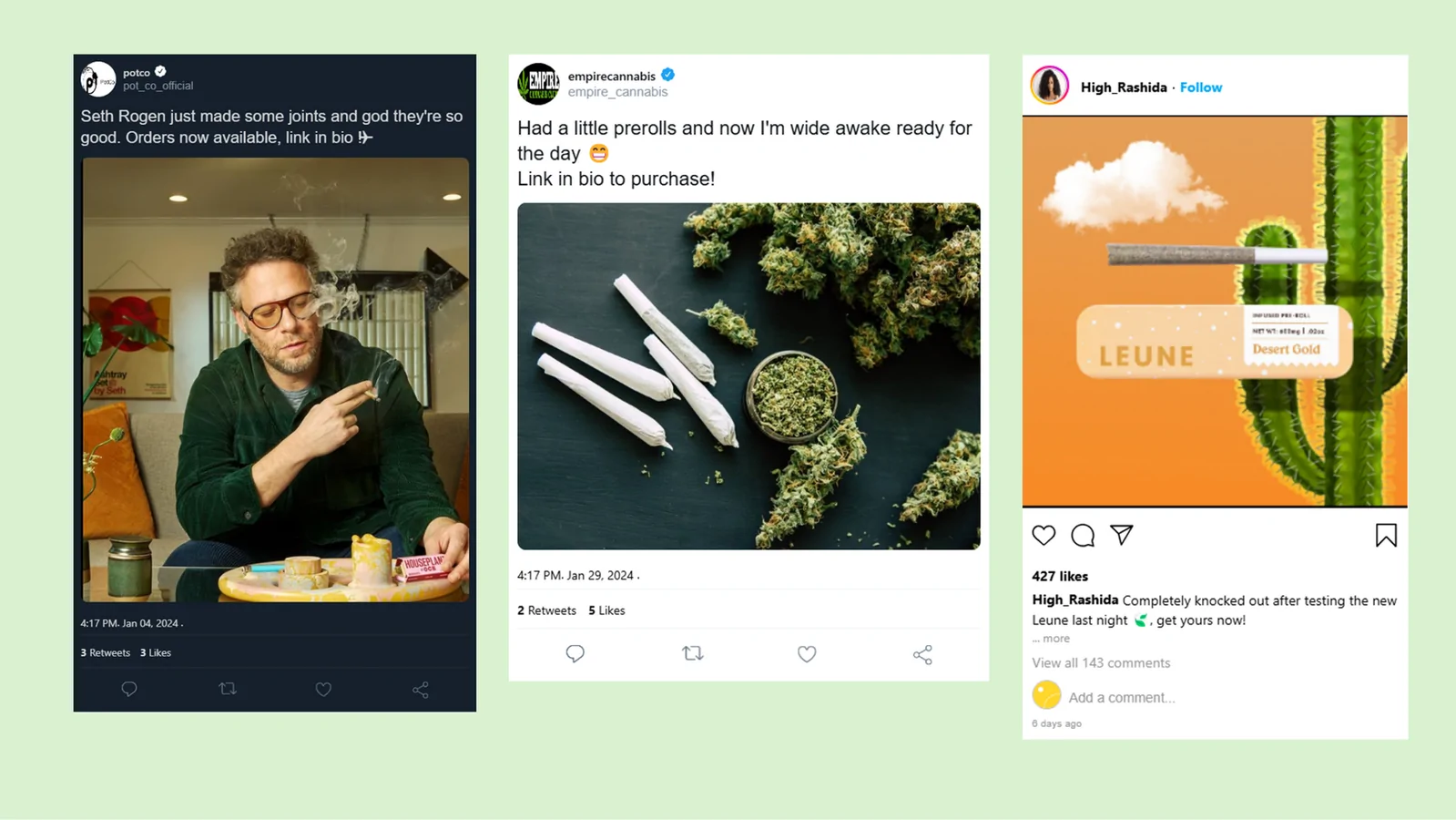
Examples of mock social media posts used in conjoint exercise. Levels displayed in figure: (*Left*) Celebrity Brand, Store, Ad + Personal endorsement, Twitter, Low engagement (*Center*) Unbranded – Plain Display, Store, Ad + Shares experience of Sativa effects, Twitter, Low engagement (*Right*) Non-celebrity Branded, Black female, Ad + Shares experience with Indica effects, Instagram, High engagement. Caption Text Annotation: (*Left*) “Seth Rogen just made some joints and god they’re so good. Orders now available, link in bio” (*Center*) “Had a little prerolls and now I’m wide awake ready for the day. Link in bio to purchase!” (*Right*) “Completely knocked out after testing the new Leune last night, get yours now!”

**Fig. 2:**
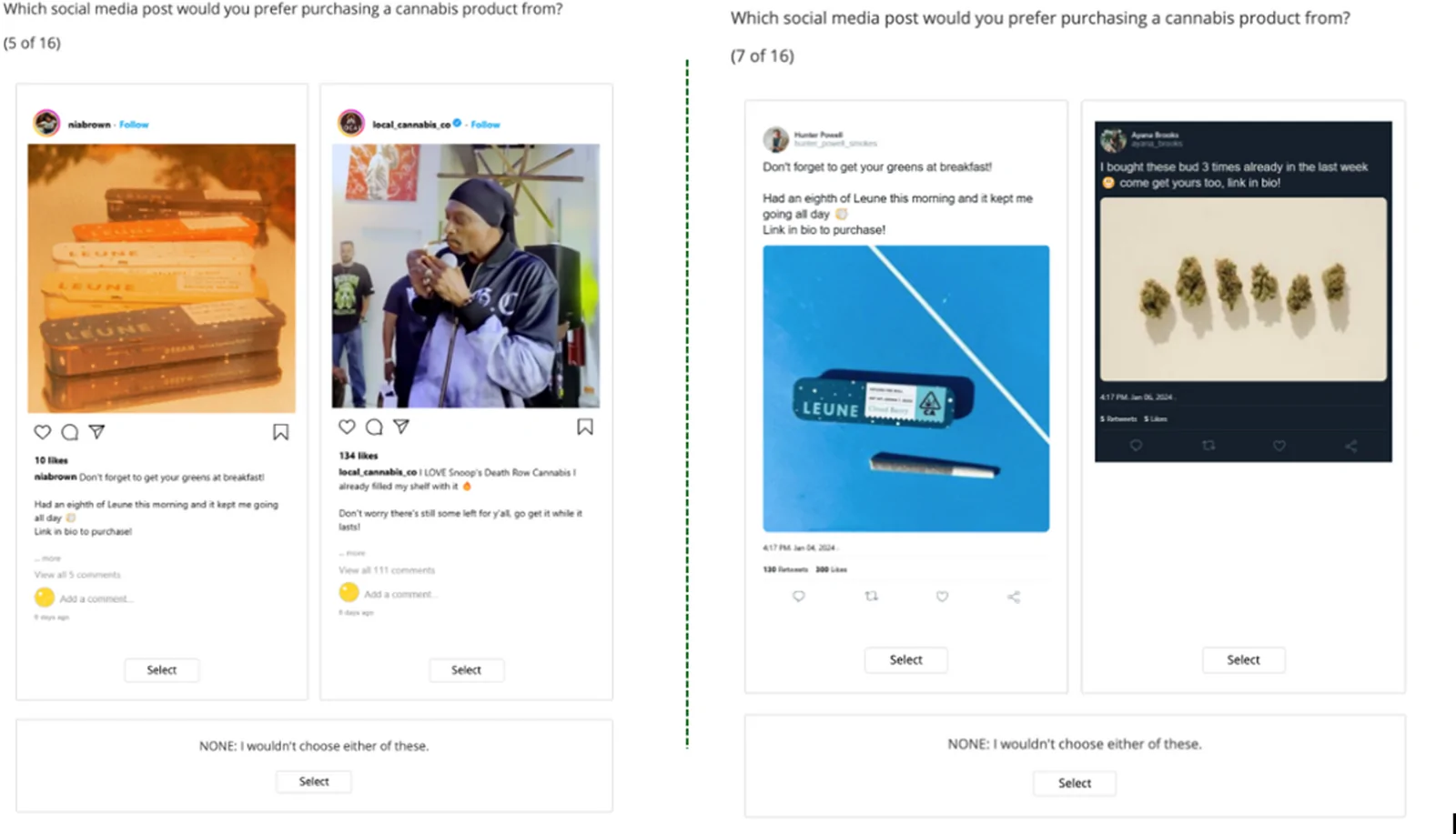
Examples of conjoint exercise tasks

The variant of conjoint analysis used in the present study is a within-subjects design called Choice-Based Conjoint (CBC), which displays multiple concepts to participants and asks them to choose their preferred option. In the current study, participants were shown two mock social media posts advertising the sale of cannabis products and asked to select the one they would be most likely to purchase from. Participants were also given the option “None: I wouldn’t choose either of these” in each exercise. We chose to only test two mock posts at a time to increase visibility of caption text in the exercise. See Figs. 1 and 2 for examples of the mock social media post and conjoint purchasing task. Each participant evaluated 32 posts in total, selectively chosen based on a balanced overlap algorithm implemented by Lighthouse Studio that ensures attribute levels are distributed equally across the exercise while allowing the same level to appear in multiple concepts within a single choice task.

Each tested level in the conjoint exercise is used as an independent variable for multinomial logistic regression modeling. Lighthouse Studios produced part-worth utility scores (i.e., beta coefficients) using hierarchical bayes estimations that account for choices from individual respondents and the sample average. Utilities for each attribute are averaged and scaled to be normalized “zero-centered diffs,” which cause the utilities to sum to 0. When interpreting results from conjoint analysis, the levels with the highest average utility scores indicate greater preference. Importantly, only utility scores of levels within the same attribute can be compared, but not across attributes. While levels cannot be directly compared across attributes, it is possible to assess which attributes overall are weighted most heavily in respondent decision-making processes by using importance scores. Importance scores are computed for each attribute by taking the difference of the range in utility values and then dividing it by the sum of the differences in ranges across all attributes.

### Survey data analysis

Attitudes and purchasing habits of cannabis use were measured using survey questions adapted from previous work examining online drug sourcing of prescription medications and controlled substances (Haupt et al. 2024, Moureaud et al. 2021). These questions were adapted for cannabis use only and measured the following: influences on cannabis perceptions, cannabis purchasing channels, product features considered when purchasing (e.g., affordability), preferred cannabis format (e.g., flower, edibles), and attitudes about legal and illicit cannabis products. See the captions in the figures from the main text and supplementary materials for the question wording used in each measure. Since past research shows associations between gender, income, education, age, urbanicity and race with cannabis use (Hasin et al. 2019, Jeffers et al. 2021, Han et al. 2017, Lim et al. 2022, Maillet 2024), these demographic factors were tested in the regression analysis. Linear regression was used to examine effects from cannabis attitudes and demographic factors on cannabis brand preference, as measured by the conjoint exercise. Specifically, utility scores for each tested level from the *Brand Display* attributed were used as outcome variables.

Consistent with previous work on illicit drug sourcing (Aldridge et al. 2018, Mackey et al. 2018, Mackey et al. 2017, McCabe and Boyd 2005, Oksanen et al. 2020, Rhumorbarbe et al. 2016), the current study defines high-risk purchasing as ever purchasing a cannabis product from at least one of the following channels: dealers/unlicensed sellers from personal networks, dealers/unlicensed sellers from online marketplaces, and the darknet. Multivariate logistic regression was conducted in order to examine potential associations between cannabis attitudes and demographic factors with high-risk purchasing behaviors. Lastly, an exploratory principle component analysis (PCA) was conducted to further examine patterns in cannabis purchasing preferences, as shown in Supplementary Table S1.

## Results

Among our sample of 253 respondents, 52.2% were male, mean age was 38 years old (SD = 11.72), 66.3% had a household income of at least $50,000 a year, and 53% had a Bachelors or graduate degree. Further, 79.8% identified as White, 11.9% Black, 7.1% Asian, and 0.8% American Indian. For ethnicity, 9.1% identified as Hispanic. When asked about frequency of cannabis use, 44.2% report using cannabis almost every day, 18.6% use 2–3 times a week, 9.9% use once a week, and 27.3% use cannabis once a month or less. All respondents participated in the conjoint exercise and survey that followed the exercise.

### Conjoint results

**Fig. 3:**
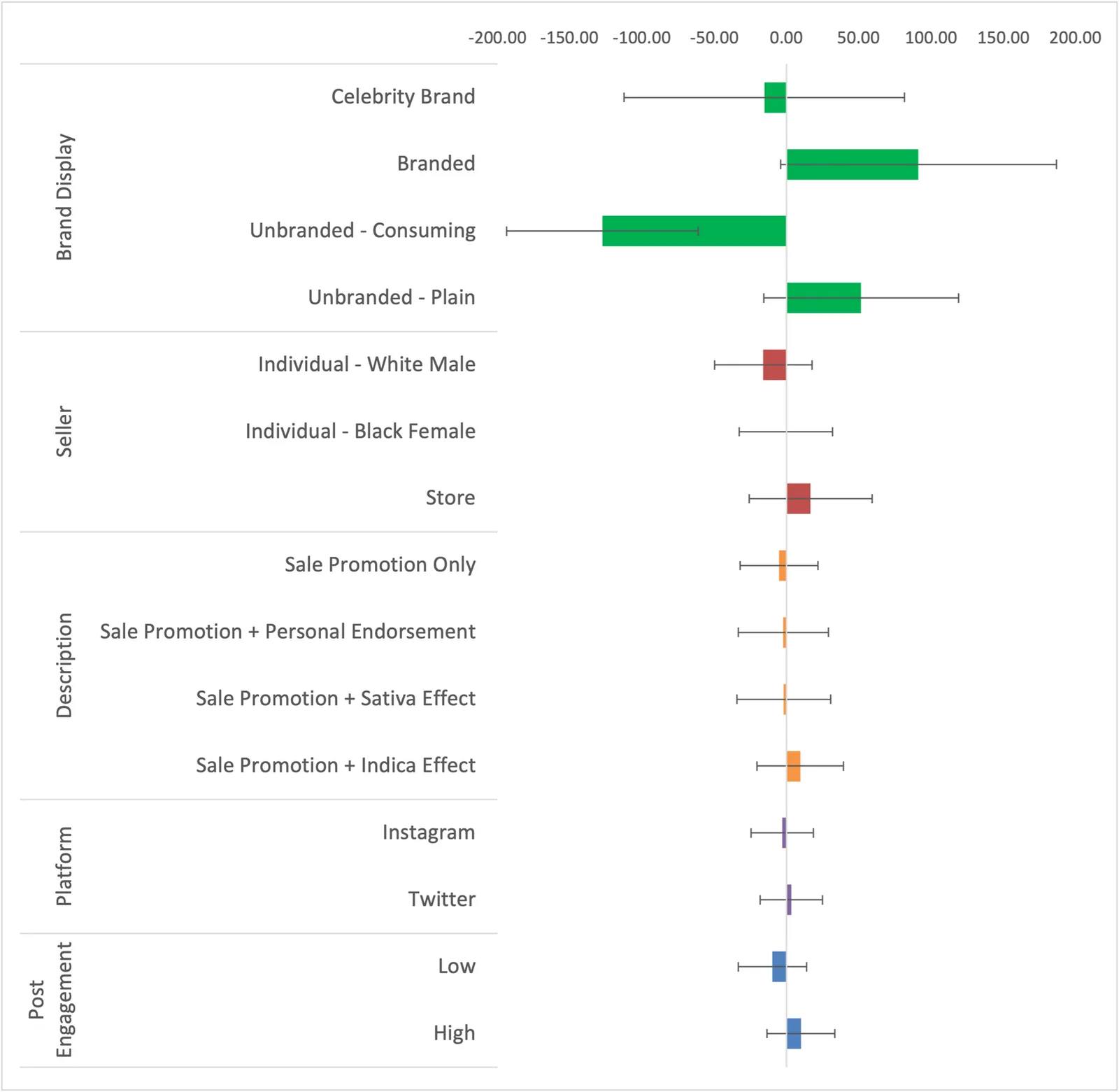
Bar chart of conjoint utility scores with error bars (± 1 standard deviation)

Table 2 shows the results for the Cannabis conjoint exercise and Fig. 3 visualizes findings as a bar chart. Among the tested attributes, *Brand Display* has the highest importance score by a large margin (56.73, SD = 15.42) while *Platform* is the lowest (6.91, SD = 5.30). Chi square results further show that the following attributes have statistically significant differences across levels when comparing the count of posts chosen in the exercise: *Brand Display* (χ = 371.191, DF = 3, *p*<.001), *Seller* (χ = 8.089, DF = 2, *p*=.018), and *Post Engagement* (χ = 7.684, DF = 1, *p*=.006).

**Table 2:** Conjoint results: average importance scores and utility scores (zero-centered diffs) for tested attributes and levels

Attribute	Average Importances (Std Dev)	Levels	Average Utilities (Std Dev)
*Brand* *Display*	56.73 (15.42)	Celebrity Brand	-15.47 (97.04)
		**Branded (Non-Celebrity)**	**91.24** **(95.36)**
		Unbranded - Consuming	-127.32 (66.26)
		Unbranded - Plain	51.55 (67.37)
*Seller*	14.89 (10.11)	Individual - White Male	-16.11 (33.78)
		Individual - Black Female	-0.47 (32.26)
		**Store**	**16.58** **(42.56)**
*Description*	13.74 (7.66)	Sale Promotion Only	-5.18 (26.97)
		Ad + Personal Endorsement	-2.26 (31.13)
		Ad + Sativa Effect	-1.99 (32.50)
		**Ad + Indica Effect**	**9.43** **(29.91)**
*Platform*	6.91 (5.30)	Instagram	-3.14 (21.57)
		**Twitter**	**3.14** **(21.57)**
*Post Engagement*	7.73 (6.69)	Low	-9.89 (23.59)
		**High**	**9.89** **(23.59)**

Bold data indicates level with the highest utility score within the attribute

Within *Brand Display*, *Branded (Non-Celebrity)* was the highest ranked level (91.24, SD = 95.36) followed by *Unbranded – Plain* (51.55, SD = 67.37). *Unbranded – Consuming* showed the lowest influence on purchase intention (-127.32, SD = 66.26). Within the *Seller* attribute, *Store* was the highest ranked (16.58, SD = 42.56) while *Individual – White Male* was lowest (-16.11, SD = 33.78). For *Description*, *Ad + Indica Effects* showed the highest preference (9.43, SD = 29.91) over descriptions that include *Sativa Effects* (-1.99, SD = 32.50) and *Personal Endorsements* (-2.26, SD = 31.13). For *Platform*, *Twitter* (3.14, SD = 21.57) was ranked higher than *Instagram*. For *Post Engagement*, *High* engagement (9.89, SD = 23.59) showed higher preference over *Low*.

### Cannabis purchasing preferences and influences

Results from our survey of conjoint respondents identified that a majority reported discussions with friends and family as an influencer of cannabis product perceptions (68.8%), as shown in Supplementary Figure S1. Online discussion forums (36.4%), scientific articles or text (28.5%), and search engine results (28.1%) were also popular influences on Cannabis product perceptions. Traditional media such as TV talk shows (6.3%) and magazines (6.3%), and discussion with teachers/professors (5.5%) were the least influential information sources. When assessing influence of specific social media platforms, Supplementary Figure S2 shows Reddit as the most popular platform for receiving Cannabis-related content (46.2%) followed by YouTube (36.4%) and Instagram (35%).

**Fig. 4:**
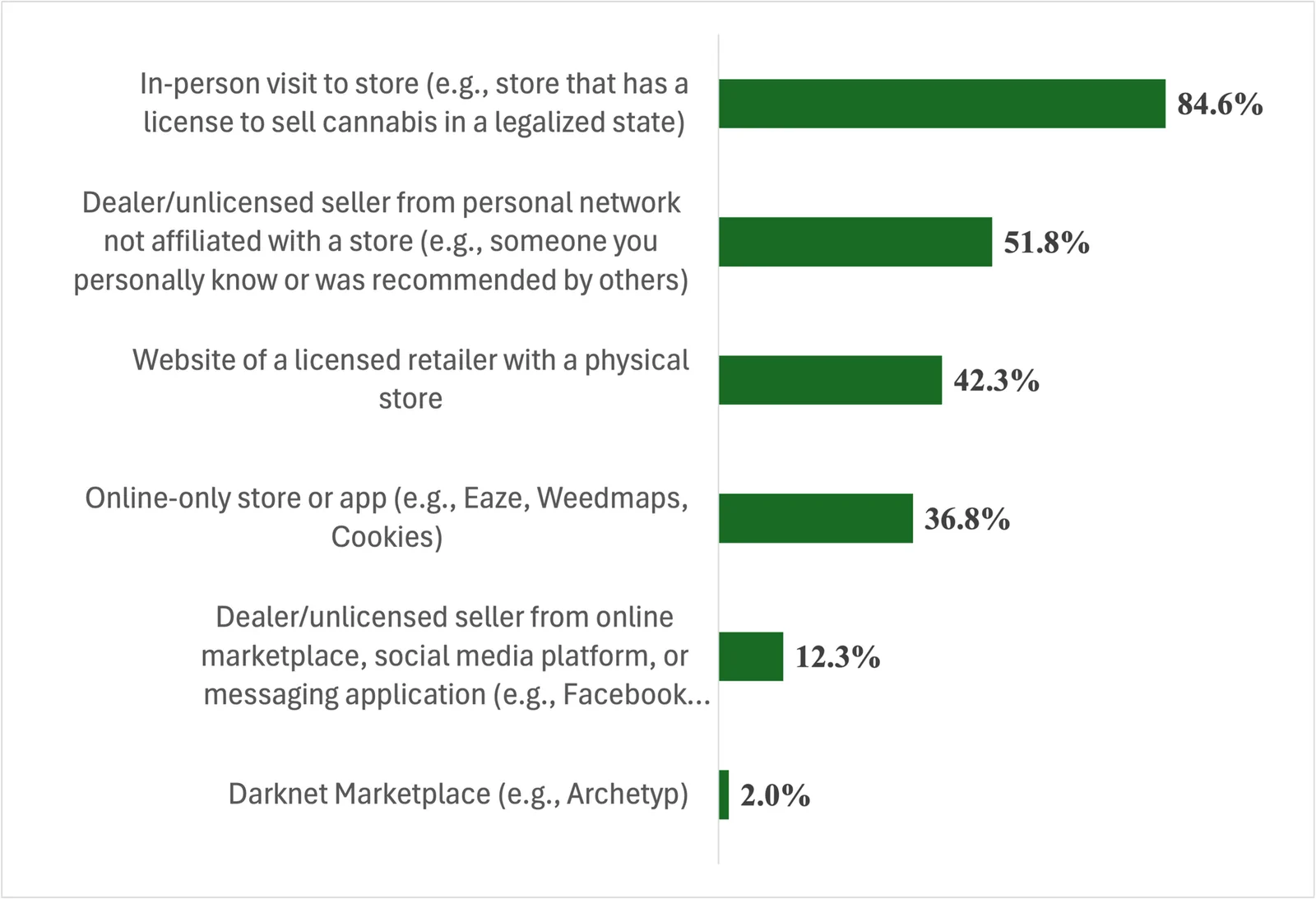
Cannabis product purchasing channels (*N* = 253). Question Text: “Which of the following have you ever used to purchase cannabis products? Please select all that apply”

Figure 4 shows Cannabis purchasing channels ever used by participants. The vast majority have purchased cannabis from in-person visits to stores (84.6%) and more than half have purchased from a dealer/unlicensed seller from their personal network (51.8%). Very few report ever purchasing from a darknet marketplace (2%). Among the total sample, 56.5% report having ever purchased cannabis from at least one high-risk channel, which includes purchases from dealers/unlicensed sellers from personal networks, dealers/unlicensed sellers from online marketplaces, and the darknet. Almost half of respondents (43.5%) report purchasing cannabis from at least one online channel. Supplementary Figure S3 shows specific platforms used to make online purchases among these users. Weedmaps was the most popular platform to purchase cannabis (22.9%) followed by social media platforms Snapchat (9.9%), Facebook Messenger (9.1%), Instagram (9.1%), and Twitter (8.7%).

**Fig. 5:**
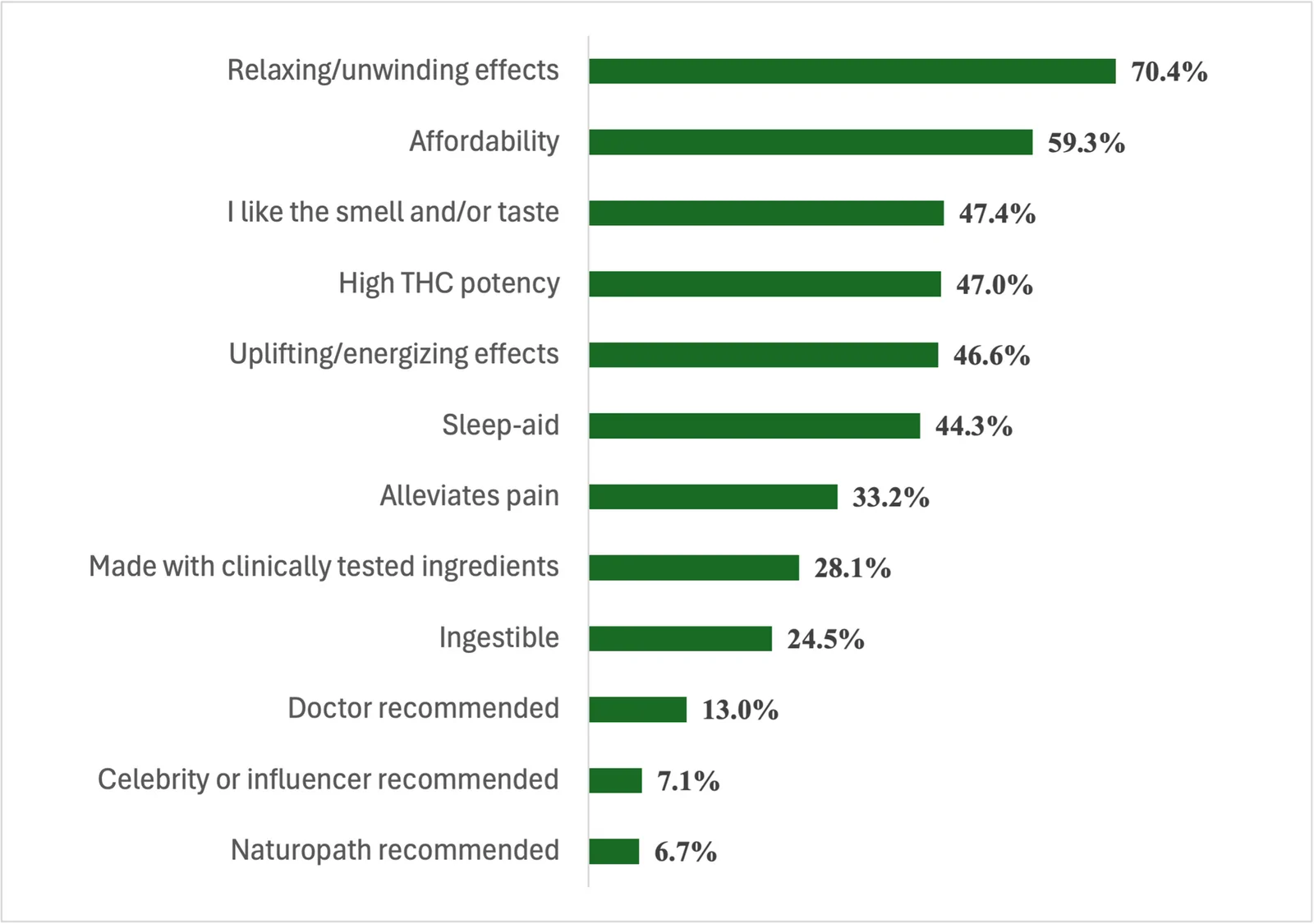
Cannabis product features (*N* = 253). Question Text: “What factors do you consider when shopping for cannabis products? Please select all that apply”

Figure 5 shows that the majority of participants consider relaxing/unwinding effects (70.4%) and affordability (59.3%) as factors when shopping for cannabis products. Other factors such as smell and/or taste (47.4%), high THC potency (47.0%), and uplifting/energizing effects (46.6%) were also considered by almost half of participants. Recommendations from doctors (13.0%), celebrities/influencers (7.1%), and naturopaths (6.7%) were the least important shopping factors.

As shown in Supplementary Figure S4, most participants prefer to purchase cannabis as edibles (70%) or as flower/herb/leaf (62.8%). Almost half of participants also purchase vape cartridges/single use vapes (45.5%). Sprays/lozenges were the least likely to be purchased (6.3%). When asked about the type of THC-based cannabis they primarily purchase, 51.8% of participants state branded cannabis products, 13.8% state unbranded products, and 34.4% report no preference for branded or unbranded products. Supplementary Figure S5 shows factors associated with brand preferences for cannabis products. The majority of participants prefer brands that offer higher quality products (59.3%), know and trust the brand name (52.2%), and has a lower price (51.4%). Offering more health benefits (19.4%) was the least selected reason for choosing a cannabis brand.

When asked about preferences for legal vs. illicit cannabis products, over half of participants agree with the statements “Legal cannabis products are better quality than illicit cannabis products” (59.3%) and “Illicit cannabis products are cheaper than legal products” (52.6%). Close to half the sample agree that “The risk to bodily health when using illicit cannabis products is high” (47.8%). Around a third of participants agree that “Illicit cannabis products are more widely available and accessible than legal cannabis products” (37.9%) and “Illicit cannabis products are as good as legal cannabis products” (31.6%).

### Multivariate regression analysis

Multivariate regression analysis was conducted to examine factors that influence cannabis branding preferences, as measured from the conjoint exercise. In Table 3, linear regression was run between attitudes towards cannabis legality, as previously reported in Section "Cannabis purchasing preferences and influences", and preferences for each tested level in the *Brand Display* attribute. Results show that believing illicit cannabis products are as good as legal products was positively associated with preferring celebrity branded products (β = 15.19, *p*=.034) and negatively associated with preference for non-celebrity brands (β = -21.87, *p*=.0017) when controlled for tested covariates. No other attitude statements showed a significant effect on brand preference.

**Table 3:** Linear regressions of cannabis brand attitudes on conjoint brand preference

	Celebrity Branded	Non-Celebrity Branded	Unbranded - Consuming	Unbranded - Plain
Legal cannabis products are better quality than illicit cannabis products	2.71 (7.35)	-4.31 (7.10)	4.23 (5.07)	-2.63 (5.19)
Illicit cannabis products are as good as legal cannabis products	15.19 * (7.15)	-21.87 ** (6.91)	9.26 (4.93)	-2.58 (5.04)
The risk to bodily health when using illicit cannabis products is high	-6.15 (5.87)	5.35 (5.67)	3.45 (4.05)	-2.65 (4.14)
Illicit cannabis products are cheaper than legal products	-0.69 (6.26)	-5.51 (6.05)	2.95 (4.32)	3.25 (4.42)
Illicit cannabis products are more widely available and accessible than legal cannabis products	-2.34 (5.51)	4.11 (5.32)	-0.49 (3.80)	-1.28 (3.89)
N	253	253	253	253
R2	0.04	0.07	0.02	0.01

** *p* < .01; * *p* < .05

Table 4 shows that education level is negatively associated with preferences for celebrity branded cannabis products (β = -16.75, *p*<.001) and positively associated with non-celebrity brands (β = 12.60, *p*=.011) when controlled for all tested variables. Those who identified as White or Asian were less likely to prefer unbranded products where consumers are shown consuming cannabis (β_White_ = -49.65, *p*=.012; β_Asian_ = -61.82, *p*=.004). Lastly, age was positively associated with preference for unbranded products that were displayed plainly (e.g., on a table) (β = 1.28) and this effect is highly significant (*p*<.001).

**Table 4:** Linear regressions of demographic factors on conjoint brand preference

	Celebrity Branded	Non-Celebrity Branded	Unbranded - Consuming	Unbranded - Plain
Gender: Male	-5.29	13.80	-12.86	4.34
	(12.76)	(12.64)	(8.59)	(8.80)
Gender: Non-binary/Non-conforming	30.83	-39.74	16.83	-7.91
	(49.69)	(49.22)	(33.45)	(34.27)
Gender: Transgender Male	-59.94	36.60	-81.59	104.92
	(97.65)	(96.73)	(65.74)	(67.34)
Education	-16.75 ***	12.60 *	1.62	2.53
	(4.93)	(4.89)	(3.32)	(3.40)
Income	-2.73	1.57	-0.65	1.81
	(4.46)	(4.42)	(3.00)	(3.08)
White (Y/N)	6.57	25.83	-49.65 *	17.25
	(29.15)	(28.87)	(19.62)	(20.10)
Black (Y/N)	10.66	-16.41	-12.70	18.45
	(31.69)	(31.39)	(21.33)	(21.85)
Asian (Y/N)	27.04	24.44	-61.82 **	10.34
	(32.04)	(31.74)	(21.57)	(22.10)
American Indian (Y/N)	18.21	76.55	-76.52	-18.23
	(71.35)	(70.68)	(48.04)	(49.21)
Area: Suburban	-12.39	10.90	2.22	-0.73
	(17.09)	(16.93)	(11.51)	(11.79)
Area: Urban	-13.62	17.72	4.26	-8.35
	(18.76)	(18.59)	(12.63)	(12.94)
Age	-0.33	-0.44	-0.51	1.28 ***
	(0.55)	(0.54)	(0.37)	(0.38)
N	247	247	247	247
R2	0.08	0.06	0.09	0.07

*** *p* < .001; ** *p* < .01; * *p* < .05

In order to test the validity of the conjoint exercise, we compared utility scores for the non-celebrity brand level and those who report primarily purchasing branded cannabis products (*n* = 131) compare to those who prefer unbranded or state no preference (*n* = 122). Mann-Whitney U test shows a statistically significant difference (W = 6408, *p*=.006) between groups, where those who primarily purchase branded products show higher preference for non-celebrity branded products in the conjoint exercise (105.03) compared to those who did not report a preference for brands (76.4). While these findings increase confidence into the validity of our conjoint exercise, it is worth noting that past purchasing behavior is self-report which is likely not as reliable as actual sales records.

Tables 5 and 6 test the same independent variables from the previous regression models but examine the likelihood of purchasing from a high-risk channel (i.e., dealers/unlicensed sellers from personal networks, dealers/unlicensed sellers from online marketplaces, and the darknet) as the outcome variable. In Table 5, a logistic regression was run between attitudes towards cannabis legality and ever purchasing from a high-risk channel. Results show that those who believe illicit cannabis products are as good as legal products (0.45 log odds, *p*=.006) and perceive illicit cannabis products as more widely available than legal products (0.27 log odds, *p*=.029) were more likely to purchase from a high-risk channel when controlled for tested covariates. However, when we ran separate regression models for each specific high-risk channel, we no longer found statistically significant associations from attitudes towards cannabis legality. This suggest effects from attitudes are less robust on high-risk purchasing tendency.

**Table 5:** Logistic regression of cannabis brand attitudes on high-risk purchase

	High-risk Cannabis Purchase
Legal cannabis products are better quality than illicit cannabis products	-0.03 (0.17)
Illicit cannabis products are as good as legal cannabis products	0.45 ** (0.16)
The risk to bodily health when using illicit cannabis products is high	-0.20 (0.13)
Illicit cannabis products are cheaper than legal products	0.03 (0.14)
Illicit cannabis products are more widely available and accessible than legal cannabis products	0.27 * (0.12)
N	253
AIC	326.49
BIC	347.69
Pseudo R2	0.16

** *p* < .01; * *p* < .05

**Table 6:** Logistic regression of demographic factors on high risk cannabis purchase

	High Risk Cannabis Purchase
Gender: Male	-0.23
	(0.29)
Gender: Non-binary/Non-conforming	-0.45 (1.10)
Gender: Transgender Male	-15.67
	(882.74)
Education	-0.38 ***
	(0.12)
Income	-0.07
	(0.10)
White	0.23
	(0.66)
Black	0.50
	(0.72)
Asian	-1.18
	(0.76)
AmerInd	-1.00
	(1.52)
Area: Suburban	-0.37
	(0.40)
Area: Urban	-0.16
	(0.43)
Age	0.02 *
	(0.01)
N	247
AIC	330.21
BIC	375.83
Pseudo R2	0.17

*** *p* < .001; ** *p* < .01; * *p* < .05

Table 6 shows a logistic regression model that examines the influence of demographic factors on tendency to make a high-risk cannabis purchase. Results show that education level is negatively associated with the likelihood of making a high-risk purchase (-0.38 log odds) when controlled for the tested demographic factors and this effect is highly significant (*p*<.001). This model also shows that age is positively associated with likelihood of making a high-risk purchase (0.02 log odds, *p*=.049). None of the other tested demographic factors showed a statistically significant effect. When running separate regression models where we separate unlicensed sellers, we find that effects remain consistent for education level (-0.37 log odds, *p*=.0011) and age (0.03, *p*=.03) for purchasing from personal networks. However, education is no longer significant when purchasing from a seller on an online marketplace and age shows a negative association (-0.06 log odds, *p*=.012). These results suggest there may be demographic differences in consumer behavior when purchasing from unlicensed sellers in a personal network compared to online marketplaces.

We also examined if cannabis use frequency was associated with higher likelihood of purchasing from a high risk channel. When tested as the only independent variable, cannabis use frequency was positively associated with a higher likelihood of purchasing from a high-risk channel (0.32 log odds, *p*<.001). This effect remains statistically significant when controlling for the demographic factors tested in Table 6 (0.30 log odds, *p*=.002), indicating that high frequency use is a prominent factor associated with high-risk purchasing.

## Discussion

This study used a conjoint exercise and survey measures to identify influential features of cannabis product ads on social media and characterize purchasing preferences among 253 respondents that have: (1) previously shopped online, and (2) have used cannabis either medicinally or recreationally. Findings support our hypothesis that product branding would be the most important attribute for intent to purchase within the conjoint exercise. When comparing average importance scores across attributes, *Brand Display* outscored all other attributes by a notable margin. This fully supports our first hypothesis (*H1*), which posits that *Brand Display* would be the highest ranked attribute. Our second hypothesis (*H2*) stating that posts featuring branded products would rank higher than unbranded is partially supported: non-celebrity branded was the most preferred level followed by unbranded product displayed on a surface. The celebrity branded level was ranked third while images of unbranded products being consumed was the least preferable level. These results are consistent with prior work showing that branding and packaging information are important factors for cannabis products (Donnan et al. 2023, Leos-Toro et al. 2021, Goodman et al. 2021), and from Haupt et al. (Haupt et al. 2024), which showed social media posts that displayed drugs in official packaging were perceived as safer compared to posts displaying drugs in a hand or using no picture at all. Within the context of social networking sites (SNS) that resemble open street drug markets, where buyers and sellers do not need to have a prior relationship to initiate a sales transaction, users may be more likely to rely on “information scents” from posts, which are defined as proximal cues on webpages (e.g., title wording, images) that indicate the value and relevance of new information to users based on their search goals (Pirolli and Card 1999). When exposed to online drug advertising, convergent findings from Haupt et al. (Haupt et al. 2024) and the present study suggest that visual displays of drug products could provide key credibility cues for users when considering a sales transaction, particularly if the product is in official packaging.

Our findings further reveal aspects of cannabis marketing that may be unique to social media compared to traditional advertising channels. According to prior research, full branded products showed higher appeal than plain packaging (Leos-Toro et al. 2021, Goodman et al. 2021), however, this previous work did not examine how product packaging was perceived within the context of a social media post, or the appeal of cannabis products displayed outside of packaging. In the present study, while non-celebrity branded products were the most preferred level, *unbranded* cannabis products displayed on a table ranked higher than celebrity branded products. The higher appeal for unbranded product displayed on a table may be attributed to the social media context, where users are accustomed to seeing content with images displaying products by themselves, such as food dishes from a restaurant or lattes from a coffee shop. Unbranded cannabis laying on a table may be appealing because it resembles this aesthetic. Our findings also suggest that explicit endorsement from celebrities for cannabis products may be more of a detriment to purchase intentions compared to unbranded products. Even though drug branding can be a key credibility signal in SNS environments resembling open markets, the present study shows that effective visual displays of cannabis products on social media can also involve products *without* branding.

The lowest level for *Brandy Display* was unbranded cannabis being consumed in social settings. This effect may also be attributed to the context of social media: while exposure to traditional advertising usually takes place in a private context where people may expect to see an ad (e.g., magazine, television), viewing ads on a newsfeed takes place within a social context, where engagement can be seen by others and users expect to see mostly content from those they intentionally follow. It could be possible that imagery of parties are more likely to evoke conformity effects in traditional ads, but may not be perceived the same way when viewed on a social media platform.

When examining demographic influences on brand preference, the regression results show that education level is negatively associated with preference for celebrity branded products, age was positively associated with preference for unbranded cannabis displayed on surfaces, and those identifying as White or Asian showed lower preference for unbranded cannabis shown being consumed. Those with higher education may have more cosmopolitan consumer tastes (Holt 1998), which could be a reason why they show lower preference for celebrity brands due to being perceived as “mass culture”. The higher preference for unbranded cannabis products among older users may be attributed to generational differences from being exposed to legal cannabis markets at a later stage of life, where stigma against cannabis use can persist among older users (Dahlke et al. 2024, Baumbusch and Sloan Yip 2022). Brand preferences associated with education and age are also consistent with findings from Leos-Toro et al. (Leos-Toro et al. 2021), which shows that cannabis packaging displaying a celebrity sponsor were perceived as being for young adults and for those with a party lifestyle. The observed effects from race may reflect social differences in cannabis perceptions and credible purchasing signals for White and Asians compared to other racial groups. Similar to age, stigmas surrounding cannabis use can also differ across racial groups (Reid 2022), which could subsequently influence purchasing preferences. Further work is needed to build on this exploratory analysis and test underlying explanations for our regression results.

Our conjoint findings further suggest that post features cueing social endorsements are more likely to signal legitimacy to potential buyers. As hypothesized in *H3* and *H5*, posts from store profiles were the most influential type of seller compared to individuals, and participants were more likely to purchase from posts with high engagement (100–500 likes, 50–250 comments/retweets) compared to low (1–10 likes, 1–5 comments/retweets). People may perceive stores as more credible since it implies there are multiple people involved in operations and higher likelihood of regulation, as opposed to individual sellers where there would be less transparency and accountability. Our findings are also consistent with results from previous work showing that cannabis users tend to purchase product from storefronts compared to individual sellers (Xing and Shi 2024, Bradley et al. 2024). Posts that received higher engagement indicate to users that multiple people saw the post and reacted (e.g., liking) to the content, signaling social approval that could encourage purchasing decisions. It is also worth noting that posts with profile pictures of Black females also ranked higher than posts from White males for purchase intent. This effect may be attributed to cultural and social associations surrounding gender and race, however, future work is needed to examine why these associations are salient for cannabis purchasing decisions. While level preferences were aligned with hypothesis *H4*, which predicted that captions describing effects from sativa and indica would be ranked higher, the *Description* attribute did not show a statistically significant difference from the Chi square results. Therefore, this hypothesis is only partially supported as the differences in preference are only directional.

A majority of users report relaxing and unwinding effects as the top factor for purchasing cannabis products (70.4%), which is consistent with results from the conjoint exercise. Most participants also report affordability as a top factor for purchasing decisions for both cannabis brands (51.4%) and products (59.3%), which is consistent with prior work showing price as an important factor for cannabis purchasing (Donnan et al. 2022, Donnan et al. 2023, Fataar et al. 2021, Robertson and Thyne 2021). Endorsements were the least influential factors for purchasing cannabis products, whether they’re from medical doctors, naturopaths, or celebrities. The lower preference for celebrity endorsements and high appeal of relaxing effects are echoed in the conjoint findings, where celebrity branded products ranked lower than unbranded cannabis, and mentions of Indica effects were the highest ranked level for post descriptions.

When assessing attitudes on product legality, a majority of participants viewed legal cannabis products as better quality while illicit products were perceived as cheaper. Our results align with previous work comparing perceptions of licit and illicit cannabis perceptions (Fataar et al. 2021, Robertson and Thyne 2021). Regression analysis further showed that believing illicit cannabis products are more accessible and as good as legal products was positively associated with making a high-risk purchase. When examining demographic factors, education level was negatively associated with ever making a high-risk purchase while age showed a positive association. This suggests that higher levels of education may mitigate the likelihood of making a high-risk purchase, which is also shown in results from prior work (Maillet 2024). The positive effect from age may reflect the fact that older cannabis users would only have had illicit channels to make a purchase before more recent changes in US state legislation, who may continue using those channels due to familiarity and loyalty to supplier (Robertson and Thyne 2021).

Findings also indicate that personal networks (e.g., friends, family, co-workers) and online forums such as Reddit are the most frequently used source of cannabis information. Our results are consistent with previous work showing the influence of family and peers on drug use habits (Harakeh et al. 2007, Hoek et al. 2022, Bahr et al. 2005, Farrell and White 1998). Public health officials aiming to mitigate harmful or illicit cannabis use among young adults should consider information campaigns targeting these online channels and relevant social groups that influence cannabis sourcing and use initiation. Since our study shows that family is a popular source of influence for cannabis perceptions, intervention programs can incorporate family members such as parents in education efforts specific to safety regarding product sourcing. Social and family-based interventions could also be intergenerational, facilitating safe sourcing discussions that cross different user ages and family roles. Our findings further indicate that potential messaging to communicate safe sourcing behavior can be promoted on the platforms Reddit, YouTube, and Instagram since they are frequently used for obtaining cannabis-related content.

PCA results, as reported in Supplementary Table S1, show higher-level groupings of product features valued by users based on: *Experience* (smell/taste, THC potency, uplifting/energizing effects, alleviates pain), *Relaxing* (sleep-aid, unwinding effects), *Health* (doctor recommended, made with clinically tested ingredients, naturopath recommended) and *Utility* (affordable, ingestible). Overall, these findings reveal how product features are associated together in purchasing decisions.

### Implications for regulatory measures

Our findings have several implications for regulatory measures on social media. The conjoint results show that posts from businesses had higher purchase intent than posts from individual sellers. Social media platforms should require verification process with a publicly visible badge for accounts marketing cannabis products to ensure the seller is legitimate and prevent illegal dealers from using a fake business name and logo to signal credibility. Our results also show that cannabis users preferred posts with higher engagement. In light of online astroturfing efforts, where entities (e.g., businesses, lobbying groups) can artificially create an impression of support for a product or policy when only limited support actually exists (Chan 2024, Haupt et al. 2021, Lyon and Maxwell 2004), there is a potential that sellers can use similar tactics to promote products. Regulatory measures used to prevent online astroturfing from AI bots and “Like farms” (Ikram et al. 2017) should be extended to detect inauthentic engagement on posts advertising cannabis products. Lastly, our findings show that those with higher age and lower education levels were more likely to purchase cannabis from a high-risk channel. This suggests that public health messaging and policies should be tailored to directly target these higher-risk groups.

### Limitations

There are multiple limitations for this study that are worth noting. One limitation is generalizability, as there may be a selection bias from Prolific. Our sample was also predominately White and skewed older than typical cannabis users, which may influence findings when considering that age and race are factors that consistently influence cannabis use. While our sample size was determined to adequately observe effects for the conjoint exercise (see OSF link for summary of the conjoint design’s statistical power), our sample is underpowered for generalizing about all cannabis users in the US, limiting the certainty of our findings from the analysis of survey questions following the conjoint exercise. Future work should recruit from additional recruitment panels to assess more national generalizations of cannabis purchasing preferences. Further, our findings on the influence from personal networks on cannabis attitudes were not able to delineate effects from family members and friends. Distinguishing between these groups can provide more nuanced insights into cannabis influencers. We also did not include manipulation checks to assess whether participants noticed differences in posts. Recording these measures could reveal important insights into cannabis purchasing tendencies by comparing post features that are consciously recognized with subliminally preferred features identified in the conjoint experiment.

As typical with conjoint analysis, tested attributes are dependent on the researcher’s decisions. Therefore, it is possible and likely that we did not test all relevant features of social media posts related to purchasing intent (e.g., price, THC potency). Due to restraints in the number of mock posts that could be created, we were only able to test 2 social media platforms (X and Instagram) and 2 variants of individual sellers (White male and Black female). Researchers interested in online cannabis purchasing should test other platforms, such as TikTok (Rutherford et al. 2022, Vassey et al. 2025), to expand on the findings from the present study. Work is also needed to examine more extensively effects from demographic characteristics of cannabis sellers on product appeal and purchasing intent. We chose White male and Black female to ensure we had two demographically distinct variants for the individual seller levels, but more combination of identities need to be tested to comprehensively examine effects from seller characteristics. Other factors such as levels of attractiveness and personal characteristics of individual sellers need to be formally tested as well.

Further, we did not stratify respondents based on the state in which they live in, where some states have legal markets for recreational use while others have no legal market. Since prior work shows that attitudes towards illicit cannabis products can vary for states with more “mature” legal markets (Robertson and Thyne 2021), it is likely that our regression results using attitudes towards cannabis legality would show greater nuance when controlling for effects from state residency. Even though we attempted to account for visibility issues of images and caption text by only testing 2 posts per conjoint task, it is possible that factors such as smaller monitor size could influence our findings. While participants can zoom into posts using built-in features on web browsers, this feature was not explicitly mentioned in the exercise instructions. Lastly, as with any simulation exercise, actual purchasing behaviors may differ from preferences shown in the conjoint exercises. Conjoint analysis typically measures decision-making processes under “ideal” situations, where preferences may be altered by confounding factors present in real-world scenarios.

## Conclusions

The expanding and diversifying cannabis product market, changing state policies and legislation, and continually evolving online environments underscore the complexity of regulating online cannabis marketing and sales. The existence of virtual marketplaces further blur legal distinctions when considering the variety of legal status for cannabis products across countries and jurisdictions. As legal cannabis markets continue to grow and invest in further marketing and expand their reach across social media platforms, it is important to design research that accounts for factors unique to online spaces to support appropriate product marketing regulation.

## Supplementary Information

Supplementary Material 1.

## Data Availability

All supplementary materials, survey data, and results from the conjoint analysis for the present study can be found at the following Open Science Framework (OSF) depository: https://osf.io/ur6ma/?view_only=673851dec544422485342b590fd36511.
