# Supplementary material for: Investigating purchasing preferences of cannabis products on social media: conjoint analysis and online survey
Source: J Cannabis Res. 2026 Jul 29;8:112. doi: 10.1186/s42238-026-00478-w (PMC13700486; doi:10.1186/s42238-026-00478-w)
Supplement: Supplementary file 1 [file 42238_2026_478_MOESM1_ESM.docx]

# Supplementary Materials

**Figure S1.** Sources of Influence on Cannabis Product Perceptions (N=253)

***Question Text***: “Which of the following, if any, have influenced your perception of cannabis products? Please select all that apply.”

**Figure S2.** Social Media Influences on Cannabis Perceptions (N=143)

***Question Text***: “Which of the following social media platform, if any, has content that has influenced your use of cannabis products? *Please select all that apply.”* ***Sample Note****:* Sample is based among those who selected an online information source as an influence of Cannabis perceptions.

**Figure S3.** Online Platforms used to Purchase Cannabis Products (N=110)

***Question Text***: “Which websites or online platforms have you ever used to purchase cannabis products? *(Select all that apply)”.* ***Sample Note****:* Sample is based among those who selected an online-based channel (n= 110).

**Figure S4.** Cannabis Product Format Preferences (N=253)

***Question Text***: “Which of the following types of cannabis products do you typically purchase? Please select all that apply.”

**Figure S5.** Cannabis Product Brand Preferences (N=253)

***Question Text***: “Which of the following best describes why you choose one brand of cannabis product over another? Please select all that apply.”

**Table S1.** Principle Component Analysis (PCA) of Cannabis Product Quality Cues

We conducted an exploratory principle component analysis (PCA) to examine which quality cues of cannabis products were most likely to be considered together. Number of components were based on eigenvector values, which resulted in 4 components. Component loadings from **Table S1** show that cues for smell and/or taste, high THC potency, uplifting effects, pain alleviation, and affordability were grouped together in *Component 1*. The label “Experience” was used for this component as these features relate to the experiences from consuming the product. For *Component 2*, being a sleep-aid and having relaxing effects were grouped together, as well as alleviates pain and being ingestible. Therefore, the label “Relaxing” was used to describe this component. *Component 3* shows that cues for being doctor recommended, made with clinically tested ingredients, and naturopath recommendation were most likely to be selected together. The label “Health” was used since these features are recommendations from health experts. Lastly, in *Component 4*, being ingestible and affordable were often selected together while celebrity or influencer recommendation was negatively associated with this component. The label “Utility” was used to describe this component because it is associated with practical features related to consumption and accessibility, while being negatively associated with celebrity endorsement.

| **Cannabis Product Quality Cues** | ***Components*** | | | |
| --- | --- | --- | --- | --- |
|  | **Experience**  ***1*** | **Relaxing**  ***2*** | **Health**  ***3*** | **Utility**  ***4*** |
| *I like the smell and/or taste* | 0.671 |  |  |  |
| *High THC potency* | 0.657 |  |  |  |
| *Uplifting/energizing effects* | 0.544 |  |  |  |
| *Alleviates pain* | 0.404 | 0.352 |  |  |
| *Sleep-aid* |  | 0.783 |  |  |
| *Relaxing/unwinding effects* |  | 0.756 |  |  |
| *Doctor recommended* |  |  | 0.71 |  |
| *Made with clinically tested ingredients* |  |  | 0.667 |  |
| *Naturopath recommended* |  |  | 0.641 |  |
| *Celebrity or influencer recommended* |  |  |  | -0.666 |
| *Ingestible* |  | 0.304 |  | 0.618 |
| *Affordability* | 0.43 |  |  | 0.538 |

***Note***: PCA with Varimax rotation. Color coding used to identify product quality cues associated with each component based on loadings.
